# Skull Base Osteomyelitis from Otitis Media Presenting as the Collet-Sicard Syndrome

**DOI:** 10.1155/2018/1407417

**Published:** 2018-03-18

**Authors:** Wong-Kein Low, Hui-Ling Lhu

**Affiliations:** ^1^Novena ENT-Head & Neck Specialist Centre, No. 04-21/22/34, Mount Elizabeth Novena Medical Centre, 38 Irrawaddy Road, Singapore 329563; ^2^Duke-NUS Graduate Medical School, 8 College Road, Singapore 169857

## Abstract

Skull base osteomyelitis can involve the jugular foramen and its associated cranial nerves resulting in specific clinical syndromes. The Collet-Sicard syndrome describes the clinical manifestations of palsies involving cranial nerves IX, X, XI, and XII. We present a rare atypical case of skull base osteomyelitis originating from infection of the middle ear and causing the Collet-Sicard syndrome. Caused by *Pseudomonas aeruginosa* and *Klebsiella pneumoniae*, this occurred in an elderly diabetic man subsequent to retention of a cotton swab in an ear with chronic suppurative otitis media. This case report illustrates the possibility of retained cotton swabs contributing to the development of otitis media, skull base osteomyelitis, and ultimately the Collet-Sicard syndrome in the ears of immune-compromised patients with chronically perforated eardrums.

## 1. Introduction

The jugular foramen (JF) is a clinically important anatomical structure located at the medial end of the skull base. Cranial nerves IX–XI pass through the jugular foramen where cranial nerve XII also lies in close proximity. Various lesions can affect the JF and its associated cranial nerves resulting in specific clinical syndromes. The Collet-Sicard syndrome (CSS) describes the clinical manifestations of palsies involving all four of these cranial nerves.

Typically, skull base osteomyelitis (SBO) or malignant otitis externa originates from the external ear canal and has been well described in the literature [1]. Less is known about atypical types of SBO, which originate from other anatomical structures such as the middle ear [2]. Although both typical and atypical SBO can involve the JF, infectious causes of CSS have been observed to be rare [3].

We present and discuss a case of CSS secondary to middle ear infection in an elderly diabetic patient who had a cotton swab retained in an ear with chronic suppurative otitis media (CSOM).

## 2. Case Report

An 82-year-old man presented to us for right ear pain and itch for about one week. He had a history of hearing loss for many years with periodic right otorrhea. He had no headaches, and his nose was asymptomatic. He was on oral medications for type 2 diabetes mellitus, hypertension, and hypercholesterolaemia. He had cardiac bypass for ischaemic heart disease 6 months before.

**Figure 1:**
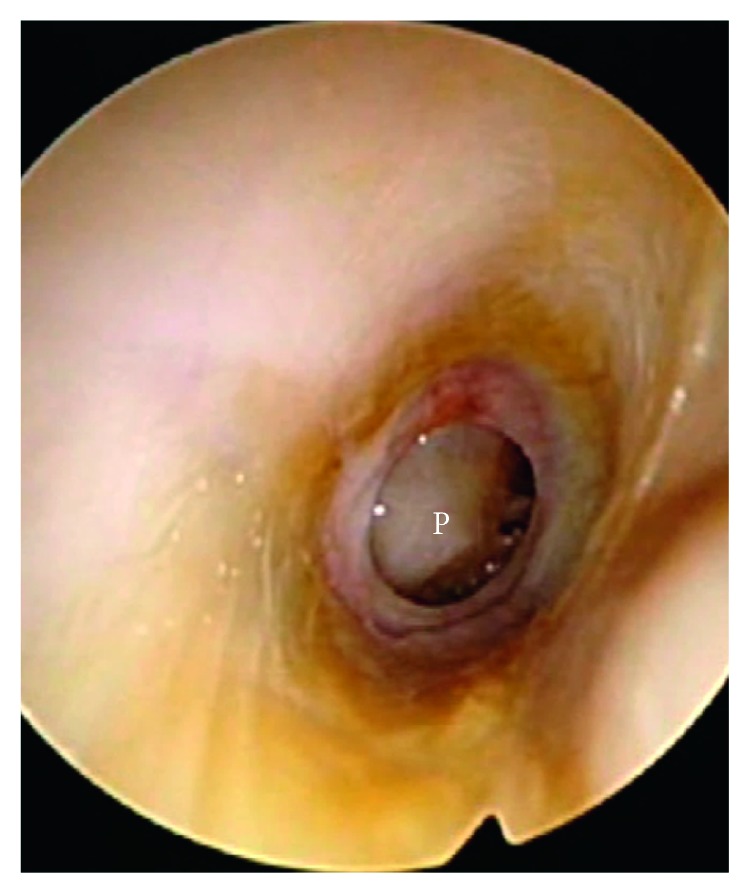
The right ear showing a large central eardrum perforation (P).

Examination of the left ear showed a healed eardrum perforation. The right ear showed a large retained cotton swab in the medial end of the external ear canal. The external ear canal skin including the bony-cartilaginous junction was not inflamed. On removing the cotton foreign body from the right ear with a pair of microforceps under microscopy, a rapid gush of copious pus was observed emanating from the middle ear through a perforation of the underlying eardrum. The eardrum perforation was large, central in position, and appeared chronic with edges that were scarred (Figure 1). Aural toilet with vacuum suction was performed, and topical ciprofloxacin eardrops were prescribed. The patient was given a 1-week follow-up review appointment which he defaulted.

The patient returned 1 month later with a new symptom of coughing while eating for 2 weeks associated with right ear pain. The symptom was worse from swallowing solids as compared to liquids, and the patient's intake was increasingly getting reduced.

He was seen earlier by a general practitioner who suspected that he might have a cardiovascular accident. An MRI of the brain was ordered which excluded an infarct of the brain.

**Figure 2:**
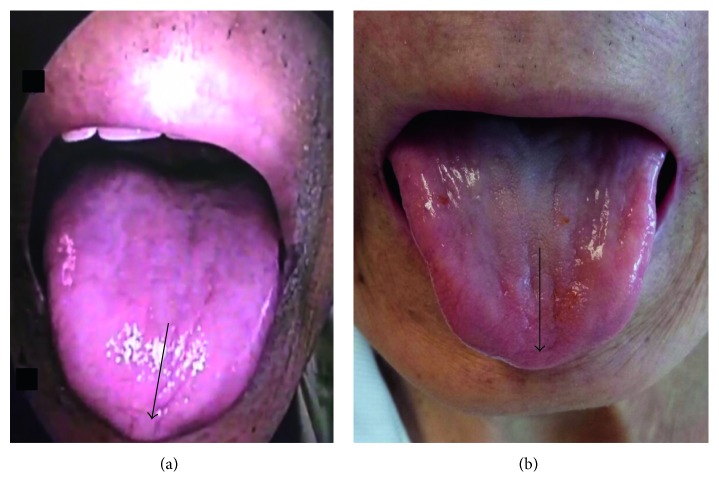
(a) Deviation of the tongue to the right on its voluntary protrusion as illustrated by the direction of the arrow (right CN XII palsy). (b) After treatment, the patient showing symmetrical protrusion of the tongue as illustrated by the direction of the arrow.

**Figure 3:**
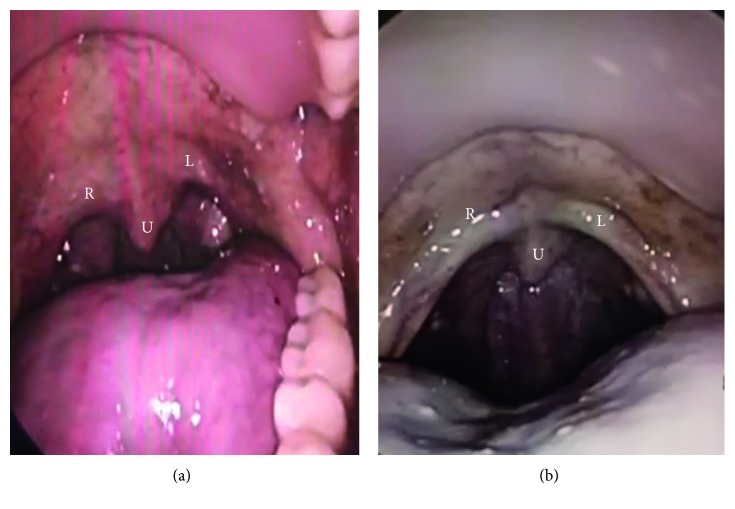
(a) Impaired elevation of the right side of the soft palate (R) as compared to the left (L) with reference to the uvula (U) (right CN X palsy). (b) After treatment, symmetrical elevation of the right (R) and left (L) sides of the soft palate with reference to the uvula (U).

The pus in the middle ear seen during the previous consult had resolved. Together with inputs from a neurologist, it was noted that the patient's tongue deviated to the right on protrusion (CN XII) (Figure 2(a)). Gag reflex on the right side was absent (CN IX), and the elevation of the soft palate on the right side was impaired (CN X) (Figure 3(a)). In addition to the reduced mobility of the right vocal cord (CN X), weakness of right sternocleidomastoid muscle (CN XI) was also observed.

Nasoendoscopy showed a slight smooth bulge on the right postnasal space. Videofluoroscopy showed moderate pharyngeal and oral dysphagia with aspiration seen on drinking fluids. A chest X-ray was reported to be normal.

**Figure 4:**
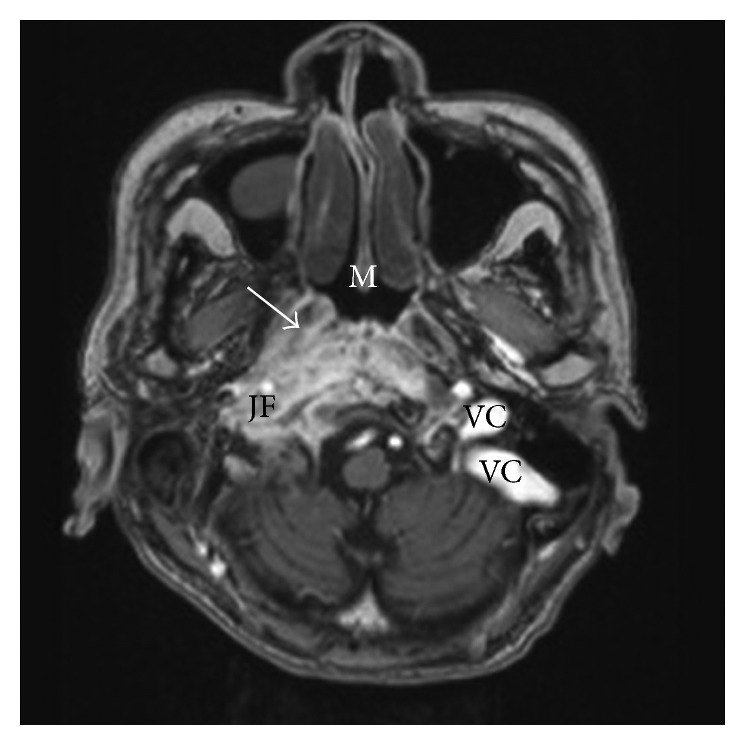
MRI scan (axial cut) of the skull base showing a large lesion involving the region of the right jugular foramen (JF). The sigmoid-jugular venous complex (VC) seen on the left side is not apparent on the right side. The lesion extends laterally beyond the jugular foramen and enhances greater than the nasal mucosa (M), and the Eustachian tubal architecture is relatively preserved (arrow).

Magnetic resonance imaging (MRI) showed a large mass with central necrosis or suppuration in the right skull base, centering at the right petrous bone and the clivus (Figure 4). It involved the right jugular foramen, the right carotid canal, the right hypoglossal canal, and the nasopharynx. The right jugular bulb and the upper right internal jugular veins were evident on the left side but not on the right. Opacification of the right mastoid air cells was noted. The differential diagnoses considered were severe skull base infection with abscess formation and neoplasm such as nasopharyngeal carcinoma.

Deep punch biopsy of the postnasal space was carried out. Histology revealed inflamed hyperplastic lymphoid tissue with no malignancy. Culture and sensitivity studies showed *Pseudomonas aeruginosa* and *Klebsiella pneumoniae*, both of which were sensitive to cefepime and ciprofloxacin. The cultures were negative for mycobacterium and fungus. Random blood glucose was raised (9.6 mmol/L), and glycated Hb suggested unacceptable glycemic control at 8.2%.

A clinical diagnosis of Collet-Sicard syndrome due to otitis media was made. He had a peripherally inserted central catheter to facilitate intravenous antibiotics. He was treated with intravenous cefepime for 6 weeks and continued with oral levofloxacin and metronidazole thereafter.

He showed progressive improvement clinically. Cranial nerve deficits reverted to normal when he was reviewed after 6 weeks of intravenous antibiotics (Figures 2(b) and 3(b)). The ear pain had resolved, and he was eating normally without symptoms. This was confirmed by biomedical indicators in that the C-protein level was reduced from the pretreatment level (107.40 mL) to <5.00 mL after intravenous treatment. The patient declined to have a repeat posttreatment MRI scan because he felt that he was already getting better.

## 3. Discussion

SBO commonly originates from the external ear canal with the typical causative pathogen being *Pseudomonas aeruginosa* [1]. In contrast, SBO originating from infections of the middle ear is rare [3]. This could partly be attributed to the fact that *Pseudomonas aeruginosa* is not a cause of acute or serous otitis media [2].

Prasad et al. opined that pus building up under tension in a tympanomastoid sealed with an intact eardrum is a prerequisite for the development of SBO [4]. This may explain why SBO is not commonly seen in CSOM where chronic perforation of eardrums is the norm. This is despite the fact that *Pseudomonas aeruginosa* is one of the commonest organisms causing active infections in CSOM [5].

Our patient was an elderly diabetic with a cotton swab retained in an ear with preexisting chronic eardrum perforation, who subsequently developed SBO. He did not have otitis externa, and the bony-cartilaginous junction of the external ear canal was normal. Upon removal of the cotton swab which was lodged in the medial end of the external ear canal, an abrupt burst of pus was seen emanating from the middle ear. There are a number of possibilities why pus could have accumulated under tension in the middle ear. Firstly, the cotton swab could have acted as a plug in preventing outflow of pus from the middle to external ear. Secondly, as a foreign body, the cotton swab is expected to aggravate any infection in the middle ear. Thirdly, inherent Eustachian tube dysfunction is often associated with a chronically perforated eardrum and, if so, could also have impeded drainage of pus from the middle ear into the nasopharynx. Culture testing of the pus grew *Pseudomonas aeruginosa* which is typically associated with SBO. In addition, the culture results also yielded *Klebsiella pneumoniae*, a microbe which had also been reported to be a cause of SBO albeit rare [6].

According to Climans et al., CSS very rarely results from otitis media [3]. Besides our case, another case of CSS originating from infection of the middle ear occurred in a 56-year-old diabetic man, but the causative pathogens in this case were methicillin-resistant *Staphylococcus epidermidis* and *Corynebacterium* [7].

Delay in diagnosis of CSS is common, partly because of its rarity and partly because of confusion with a primary intracranial event [7]. As illustrated by our case, the neurological manifestations of CSS could be mistaken to be the result of a cardiovascular accident. It is worth noting that cardiovascular accidents involving the brainstem rarely result in isolated palsies of the lower cranial nerves. Such patients are more likely to have additional neurological deficits as a result of involvement of other brainstem structures such as the spinothalamic, spinocerebellar, and pyramidal tracts [8].

Interpretation of the radiological features of CSS could be challenging. Handley et al. reported a case of CSS not caused by SBO but by idiopathic thrombosis of sigmoid-jugular sinus [9]. In our patient, MRI revealed right jugular bulb and upper internal jugular vein obliteration (Figure 4). Although this could suggest venous thrombosis, it could also be due to mere external compression by the surrounding diseased tissues. In any case, even if venous thrombosis has occurred, the extensive skull base involvement makes it more likely to be a secondary event than as a primary cause of the disease.

The extensive SBO lesion demonstrated by MRI (Figure 4) could also be confused with that of advanced nasopharyngeal carcinoma, a cancer common among Chinese in our local population. Goh et al. suggested that lateral extension, increased T2 signal, lack of architectural distortion, and enhancement greater than or equal to mucosa could be used as differentiating features [10]. As illustrated in Figure 4, SBO has the tendency to extend laterally, to lack architectural distortion, and to demonstrate greater enhancement than mucosa. However, as illustrated by our case, tissue biopsy is ultimately often indicated not only to exclude nasopharyngeal carcinoma but also for culture and sensitivity testing of the causative pathogens.

## 4. Conclusion

SBO can affect the JF resulting in CSS, a potentially life-threatening condition. Although rare, awareness of SBO as a cause of CSS is important because a delay in its diagnosis is common. Besides the typical type of SBO originating from the external ear canal, atypical types of SBO can originate from other sites such as the middle ear. This case report illustrates the possibility of a retained cotton swab contributing to the development of otitis media, SBO, and ultimately CSS in the ear of an elderly diabetic with a chronically perforated eardrum.
